# Simplification of Radiographic Technic

**Published:** 1918-10

**Authors:** 


### ROENTGENOLOGY

***Simplification of Radiographic Technic.*** By Dr. FORNARIO, Read at Meeting of the Society for Medical Propaganda in war, Sept. 23, 1917 (Translation).

The writer gives the following particular concerning a method for obtaining a radiographic image directly impressed and fixed on papers. This paper is first rendered impermeable to water and then spread with a fluorescent layer of gelatine containing bromide of silver. The development and the fixing are the same as for plates.

Owing to the hardness of the sensitive layer, it is necessary to prolong the developing until the whole sensitized layer is completely affected and the image comes out clearly. It develops more clearly if 15-16 grams per liter of bromide be added to the solution. For the same reason it is better to prolong the fixing.

With these methods the radiographic image on paper will have the same strength and clearness and good contrast of details as with plates. The picture once obtained does not change.

With this paper it is possible to obtain a radiographic picture on several layers of the paper superimposed, using 16 to 20 at a time, with a very slight difference of clearness between the first and the last.

The image can be obtained on a plate and on one or several pieces of paper at one exposure. The layers of paper should be put over the plate because the plate diminishes the intensity of the rays, while the layers of paper leave on the plate only a slight impression of their presence, without modifiying greatly the image on the plate. If one does not use the compressor (which limits the field), it is well to have the paper the same dimensions as the plate.

The article is accompanied with many half-tone illustrations of the good results obtained by this method.

The writer sums up the advantages of his methods as follows :

(1) A direct image.

(2) A greater facility, rapidity, and simplification of the technic.

(3) Economy in expense, time, and the ability to take several copies contemporaneously.

(4) The results, except in special contingencies, are equal to those on plates in most cases.

(5) Advantages in the manipulation and in the transportation of a pliable instead of a fragile plate.

(7) Finally, the possibility of writing on the paper short notes or a history of a case.

The experiments were made by the writer in the radiographic room of the Institute Jolando, in the Territorial Hospital of the Italian Red Cross. The paper is now produced at the Tensi factory in Milan.

